# The Effect of Item Similarity and Response Competition Manipulations on Collaborative Inhibition in Group Recall

**DOI:** 10.1038/s41598-017-12177-x

**Published:** 2017-09-20

**Authors:** Huan Zhang, Yao Fu, Xingli Zhang, Jiannong Shi

**Affiliations:** 1 CAS Key Laboratory of Behavioural Science, Institute of Psychology, Beijing, China; 2 Department of Psychology, College of Education Science, Tianjin Normal University, Tianjin, China; 3 Academy of Psychology and Behavior, Tianjin Normal University, Tianjin, China; 4 University of Chinese Academy of Sciences, Beijing, China

## Abstract

Collaborative inhibition refers to when people working together remember less than their predicted potential. The most common explanation for this effect is the retrieval-disruption hypothesis during collaborative recall. However, several recent studies have obtained conflicting results concerning this hypothesis. In the current study, item similarity was manipulated in Experiment 1 by requiring participants to study overlapping or non-overlapping unrelated wordlists. The unstructured instructions were then manipulated during a turn-taking recall task between conditions. The results showed that collaborative inhibition occurred for both overlapping and non-overlapping conditions. Subsequently, response competition during collaborative recall, in addition to item similarity, was manipulated in Experiment 2, and the results showed that when collaborative group members were instructed to recall in turn and monitor their partner’s recall (the medium- and high-response-competition conditions), collaborative inhibition occurred. However, no such effect was shown when collaborative group members were instructed not to communicate with each other, but to simply recall in turn while in a group (low-response-competition condition). Together, these results suggest that the conflicts between the findings of the aforementioned studies were probably caused by differing instructions, which induced response competition in collaborative settings. Aside from retrieval-disruption, other possible mechanisms underlying collaborative inhibition were also discussed.

## Introduction

For over a century, cognitive psychologists have focused on individuals’ memory performance^1^. However, in real life, people encounter numerous opportunities to communicate with others in terms of recall behaviour. This common process of recalling information is known as collaborative memory. How does collaboration influence people’s memory performance? What are the mechanisms that underlie this process?

Researchers have used the classical collaborative memory paradigm to explore the effects of collaboration^2^. The process of this paradigm is as follows: in the encoding phase, participants usually study items individually. Then, after a filled delay, participants are divided into two kinds of groups. One is the so-called ‘collaborative group’, which includes two or more members who recall together, and the other groups, the ‘nominal group’, includes an equal number of members who recall alone. In a typical experiment, the effect of collaboration is measured by comparing the number of items retrieved by the collaborative group with the number of non-redundant items retrieved by the nominal group. Researchers also typically measure the post-collaboration effect, usually via an individual recall task performed after collaboration^3,4^.

Weldon and Bellinger^4^ first demonstrated that, in the retrieval phase, individuals who work together as a collaborative group perform much more poorly than the same number of people who recall individually. This phenomenon is called *collaborative inhibition*. The most prominent theoretical explanation for collaborative inhibition is the retrieval-disruption hypothesis^3^. According to this hypothesis, during collaboration each individual’s idiosyncratic organization of information can be disrupted while they listen to the recall of other group members. This in turn damages the individual’s (and, thus, the group’s) recall performance^3,5–12^. Furthermore, this inhibition effect disappears in the subsequent individual recall task, when individuals’ retrieval organization is not disrupted by the presence of others^11,13^.

One of the main ideas of the retrieval-disruption hypothesis is that, during the collaborative recall phase, hearing another person recalling non-overlapping rather than overlapping items is less disruptive to an individual’s organizational strategy^3^. Until now, two studies have confirmed this viewpoint of the hypothesis. In Basden *et al*.’s^3^ Experiment 3 with categorized wordlists as materials, item similarity was manipulated during the retrieval phase so that each participant in a collaborative group was asked to recall from either all studied lists (overlapping condition) or separately studied lists (non-overlapping condition). The collaborative groups’ recall performances were then compared between conditions with the nominal groups’. Another study used emotional pictures as studied materials to further examine the influence of item similarity on collaborative inhibition^14^. A marginally significant interaction between item similarity and retrieval condition was observed in both studies. Thus, both studies concluded that non-overlapping information was less susceptible to collaborative inhibition than overlapping information, which supports the retrieval disruption hypothesis. This indicates that items recalled by others may be more or less disruptive to another’s memory depending on how similar they are to the other person’s recalled items^3,14^.

S﻿everal studies have used similar experimental designs and procedures, but surprisingly failed to support this viewpoint of retrieval-disruption^7,15,16^. In a study involving adults, Meade and Gigone^16^ found significant collaborative inhibition in both overlapping and non-overlapping conditions, the interaction between item similarity and retrieval condition was non-significant, and there were no differences in the magnitude of collaborative inhibition between conditions (Experiment 2). Consistent with this conclusion, Gummerum *et al*.^15^ recruited nine-year-old children and observed that an equal magnitude of collaborative inhibition occurred in both overlapping and non-overlapping conditions. In another study involving adults, researchers used entirely non-overlapping items (but no overlapping condition) as studied materials, in accordance with their research aims, and observed persistent but attenuated impairment of collaborative participants’ recall performance^7^.

Although all the above studies insisted that their manipulations of non-overlapping conditions maximally excluded the role of retrieval-disruption during collaborative recall, they found different results for the occurrence of collaborative inhibition. Basden *et al*.’s study^3^ is the most cited and important evidence that supports the retrieval-disruption hypothesis’ role in collaborative inhibition. However, even when similar variables and manipulations to this study were applied in the aforementioned studies, completely conflicting results were consistently obtained^3,7,14–16^. It is important to determine the real reason that affects the occurrence of collaborative inhibition in the aforementioned studies. Could collaborative inhibition disappear when we retrieve totally different items in a collaborative group?

Considering that Barber *et al*.’s^7^ study did not include an overlapping condition compared to the non-overlapping conditions, and another study mentioned above used emotional pictures as materials which might have introduced other confounding factors^14^, we only focused on the studies which used the wordlists as materials and manipulated item similarity to detect its influence on collaborative inhibition. The relevant studies mentioned above mostly used categorized wordlists as the study materials, but this manipulation could also create disruption to the others’ organizational strategy in a non-overlapping collaborative recall condition (i.e. cross-cuing or category-switching^16^). Considering this, we used unrelated wordlists as studied materials among conditions in our experiments. Additionally, regarding the collaborative recall of overlapping vs. non-overlapping items, one interpretation of the retrieval-disruption hypothesis is that, if the items studied by a member are different from those of their group partners, the items will not form part of the partners’ strategies. Consequently, they should be less disruptive, resulting in smaller or no collaborative inhibition effects for non-overlapping items^3^. To examine this viewpoint on retrieval-disruption, we explored collaborative recall performance for information that was studied by all group members (overlapping condition) and information that was studied by a subset of group members (non-overlapping condition) in Experiment 1. These manipulations were all intended to maximally eliminate the possibility of recalled items disrupting others’ organization strategy in non-overlapping condition during collaborative recall. Therefore, the results we obtained here would more strictly examine the core viewpoint of the retrieval-disruption hypothesis considering item similarity variables.

To examine the core viewpoint of the mechanism raised by Basden *et al*., we used a similar manipulation during collaborative recall, which required participants to recall in turn. However, no other structured instructions were given to the participants (i.e. discussion of recalled items was not forbidden) in that phase, which is somewhat different from Basden *et al*.’s study. This was because we wanted to improve our results’ ecological validity, as people often discuss and judge others’ ideas or recalled items in real life.

Based on the above analyses, it is important to continuously repeat, retest, and modify the established hypothesis by considering the growing conflicting evidence. To detect the possible reason underlying the different results, as well as to amend the retrieval-disruption hypothesis underlying collaborative inhibition, Experiment 1 presented here used unrelated words as studied stimuli to investigate the effect of item similarity on group recall performance. If our results showed that collaborative inhibition occurred in the overlapping condition but not in the non-overlapping condition, this would indicate that the theory of the retrieval-disruption hypothesis, which was raised by Basden *et al*.^3^, is reliable, or we can say that the retrieval-disruption hypothesis is stable by manipulating the item similarity variable. If our results showed that collaborative inhibition occurred in both the overlapping and non-overlapping conditions, this would indicate that the theory of retrieval-disruption should be amended, or other potential factors could also induce collaborative inhibition. If our results showed that collaborative inhibition did not occur in the overlapping condition, regardless of the non-overlapping condition, this would further question the role of the retrieval-disruption hypothesis underlying collaborative inhibition.

## Experiment 1: Effects of item similarity on collaborative inhibition

### Method

#### Participants

A total of 56 Tianjin Normal University undergraduates and graduates (mean age: 22.13 ± 1.90 years old) participated in this study. All participants were paired equally (28 dyads of strangers) and, as much as possible, randomly assigned to the collaborative or nominal condition (see Table 1). That is, we initially assigned participants equally and randomly. For example, in Experiment 1, there were 12 dyads of participants in each group. Furthermore, the last four pairs were assigned to the collaborative group, because we worried that some participants may not have fully understood the rules of the experiment and would need to be excluded. Moreover, the nominal group was relatively easier to recruit for than the collaborative group; hence, we prepared more collaborative groups. Ultimately, there were no excluded participants, so the N’s were different between groups in the experiments. The same participant assignment method was used in Experiment 2. All experiments in the current study, including any relevant details, were approved by the Ethics Committee of the Institute of Psychology at Chinese Academy of Sciences, and all experiments were performed in accordance with relevant guidelines and regulations. All participants gave informed consent prior to the experiments, in accordance with the Declaration of Helsinki.

**Table 1:** Mean proportion of overlapping and non-overlapping items’ recall errors as a function of nominal or collaborative recall in Experiment 1.

	Nominal recall	Collaborative recall
Number of groups	12	16
Overlapping	0.13 (0.08)	0.12 (0.09)
Non-overlapping	0.08 (0.05)	0.04 (0.04)

*Note*. Figures in parentheses denote standard deviations (*SD*).

#### Design

Experiment 1 consisted of a 2 (item similarity: overlapping, non-overlapping) × 2 (retrieval condition: nominal, collaborative) mixed-participants design. Item similarity was a within-participants variable and retrieval condition was a between-participants variable (previous research shows that the between-participants design of the retrieval condition is acceptable in a collaborative recall paradigm^3,15,16^). The primary dependent variable was the mean proportion of items correctly recalled in group- and individual-recall tasks.

#### Materials

We selected 90 unrelated neutral words from the Chinese Affective Words System^17^: 30 verbs, 30 nouns, and 30 adjectives. Each Chinese word was two characters in length, and had a frequency of 8–190 occurrences per million words. According to the Chinese Dictionary of Modern Chinese Frequency^18^, these words were medium and low frequency words. The words were put into three 30-word lists (10 verbs, 10 nouns, and 10 adjectives) that were matched in affect, excitement, dominance, familiarity, strokes in the first character, and strokes in the last character. In Experiment 1, wordlist 1 was only used in the overlapping condition, while wordlists 2 and 3 were used in the non-overlapping condition. The words used in Experiment 1 are listed in the Supplementary material in Chinese, along with their English translations.

Before the formal experiments, another ten undergraduates were asked to remember and recall all three wordlists serially and individually. Results showed that the mean proportions correctly recalled among these wordlists were statistically non-significant (*M*_1_ = 0.30, *M*_2_ = 0.30, *M*_3_ = 0.28, *F* (2, 18) = 0.28, *p* = 0.76), which indicated that the differences of recall performance in the formal experiments were not due to the materials themselves but to the specific experimental manipulations.

#### Procedure

(a) Encoding: Participants sat at separate computers and individually studied one of the lists in preparation for a memory test. Words appeared one at a time for 2000 ms in the centre of the screen, with an inter-stimulus interval (ISI) of 1000 ms. In our three wordlists, no two consecutive words were of the same kind (i.e. noun, verb, adjective, noun, adjective); this was to remove the possible effect (i.e. remote association) caused by similar kinds of words. Consistent with most collaborative inhibition research, participants in the collaborative groups were told that they would be tested later as a group. Participants in the collaborative groups were told that some items overlapped between group members and some items did not (individual participants were not given these instructions because they were not relevant). This was intended to provide a distinction from other paradigms (such as memory-conformity paradigms^16^). (b) Filled delay: A mathematical filler task was applied to prevent rehearsal using short-term memory. All participants were asked to calculate a simple equation (addition and subtraction within two-digit numbers) presented on the screen and write their answers. Equations appeared one at a time for 2500 ms, and ISI was 1500 ms. The filler task lasted for 60 s. (c) Group (nominal or collaborative) recall task: Both groups of participants were asked to recall aloud the words they learned and to write them down in any order by themselves. The time allotted for recall in both groups was four min. In the collaborative group, participants were asked to recall in turn with unstructured instruction (i.e. discussion of learned items was not forbidden). In the nominal group, participants recalled words at their own pace. The recall paper was collected once the recall ended. (d) Subsequent individual recall task: Immediately following the group recall tasks, all participants were given four min. to freely recall the words learned from the encoding session. Regardless of condition, participants worked individually to recall aloud and write down words they remembered from their *own* study list.

After one block, participants would have one min. to rest before beginning a new block following the same steps above (i.e. collaborative group members first studied overlapping items and then studied non-overlapping items, or vice versa). The two blocks were counterbalanced in Experiment 1. All recall sessions were tape-recorded and verbal encoding occurred afterwards.

### Data availability

The datasets analysed during the current experiment are available from the corresponding author on reasonable request.

## Results

### Group recall performance

**Figure 1:**
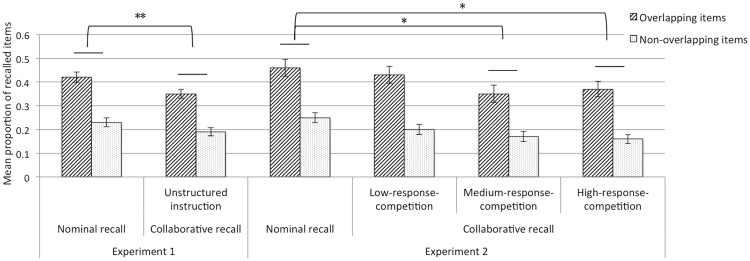
Mean proportion of overlapping and non-overlapping items correctly recalled as a function of recall conditions in both experiments (^*^ *p* < 0.05, ^**^ *p* < 0.01, ^***^ *p* < 0.001).

The mean proportion of overlapping and non-overlapping items correctly recalled in groups (nominal or collaborative) is presented in Fig. 1. A 2 (item similarity: overlapping, non-overlapping) × 2 (retrieval condition: nominal, collaborative) mixed-factor ANOVA on recall showed that the main effect of item similarity was statistically significant, which showed that overlapping items were better recalled than non-overlapping items, *F* (1, 26) = 77.39, *p < *0.001, partial η^2^ = 0.75. The main effect of retrieval condition was also statistically significant, which revealed significant collaborative inhibition among conditions, *F* (1, 26) = 9.46, *p* = 0.005, partial η^2^ = 0.27. However, the interaction between item similarity and retrieval condition was non-significant, *F* (1, 26) = 0.97, *p* = 0.33.

The use of parametric testing such as ANOVA when using proportions as a dependent variable in the current study was proved to be valid because we conducted Levene’s tests after all ANOVAs, considering that assumptions become especially serious when responses are in the lower or upper 20%. A Levene’s test on correct recall in groups showed that the deviations in the overlapping condition were symmetrical, *F* (1, 26) = 0.003, *p* = 0.958, which results could be considered as normally distributed (Skewness = 0.144, Kurtosis = −0.556); and the deviations in the non-overlapping condition were symmetrical, *F* (1, 26) = 2.70, *p* = 0.112, which results could also be considered as normally distributed (Skewness = 0.098, Kurtosis = −0.795).

### Group recall errors

Based on the analysis of correct recall among the different conditions, we also focused on recall errors during the group recall phase, because previous studies demonstrated that collaborative recall could prune errors compared to the nominal condition^16,19,20^. Thus, the mean proportion of recall errors for the different conditions, or for the items recalled that were not actually presented in the study list, is shown in Table 1. A 2 (item similarity: overlapping, non-overlapping) × 2 (retrieval condition: nominal, collaborative) mixed-factor ANOVA revealed a main effect of item similarity, *F* (1, 26) = 31.38, *p < *0.001, partial η^2^ = 0.55. However, there was no main effect in the retrieval conditions, *F* (1, 26) = 1.05, *p* = 0.31. There was also no interaction between item similarity and retrieval condition, *F* (1, 26) = 0.52, *p = *0.48.

A Levene’s test on recall errors in groups showed that the deviations in the overlapping condition were symmetrical, *F* (1, 26) = 0.127, *p* = 0.725, which results could be considered as normally distributed (Skewness = 0.705, Kurtosis = −0.788); and the deviations in the non-overlapping condition were symmetrical, *F* (1, 26) = 0.573, *p* = 0.456, although, the errors in the non-overlapping condition were somewhat non-normally distributed (Skewness = 1.650, Kurtosis = 2.843). Thus, we continually conducted a Friedman test on recall errors between overlapping and non-overlapping conditions, and the results showed that there was a statistically significant difference between groups, *χ* ^2^ = 24.143, *p* < 0.001, which was consistent with the parametric testing results. As a result, the parametric testing used above was considered to be valid.

### Subsequent individual recall performance

The primary outcome of interest in the current experiment was the effect of item similarity on collaborative inhibition; in addition, we included a subsequent individual recall test after collaboration. We did this because, according to the retrieval-disruption hypothesis, collaborative recall often benefits later individual retrieval^21^. This benefit occurs because in the subsequent individual recall task, individuals’ retrieval organization is not disrupted by the presence of others^11,22,23^; furthermore, collaborative recall can serve as a second study opportunity, re-exposing participants to items that they had forgotten but that their group members recalled^21^.

Building on this, we tested how correct recall changed from collaborative recall to subsequent individual recall as a function of retrieval condition. We collapsed the overlapping and non-overlapping nominal group members’ subsequent individual recall performance because of its ‘artificial nature’, so we conducted one-way ANOVA on the correct recall in the subsequent individual recall test among the overlapping collaborative group members, the non-overlapping collaborative group members, and the nominal group members. The results showed that there was a main effect among conditions, *F* (2, 109) = 3.45, *p* = 0.04. Further, there was no significant difference between the subsequent individual recall performance of the overlapping collaborative group members (*M*_*overlapping*_ = 0.25, *SD*_*overlapping*_ = 0.09) and the nominal group members (*M*_*nominal*_ = 0.22, *SD*_*nominal*_ = 0.08), *p* = 0.39; there was no significant difference between the subsequent individual recall performance of the non-overlapping collaborative group members (*M*_*non-overlapping*_ = 0.19, *SD*_*non-overlapping*_ = 0.08) and the nominal group members (*M*_*nominal*_ = 0.22, *SD*_*nominal*_ = 0.08), *p* = 0.53; these results demonstrated that the collaborative inhibition effect disappeared in the subsequent individual recall task^11^. However, there was a statistically significant difference between the subsequent individual recall performance of the overlapping collaborative group members and non-overlapping collaborative group members, *p = *0.03. This significant difference further confirmed the effect of re-exposure and re-learning on the overlapping collaborative recall phase, and this effect would benefit collaborative-group-members’ subsequent individual recall performance.

A Levene’s test on correct recall in subsequent individual recall showed that the deviations among conditions were symmetrical, *F* (2, 109) = 0.598, *p* = 0.552, which results could be considered as normally distributed (Skewness = 0.147, Kurtosis = −0.263).

## Discussion

In Experiment 1, even with our manipulation of the similarity of the studied items, the collaborative inhibition effect occurred in both the overlapping and non-overlapping conditions. These results are not consistent with Basden *et al*.’s^3^ conclusion, which stated that items recalled by others can be more or less disruptive to another’s memory depending on how similar they are to the other partner’s recalled items, and the researchers also insisted that the retrieval-disruption hypothesis is the sole-mechanism of collaborative inhibition. Although most studies have supported the retrieval-disruption explanation, collaborative inhibition with non-overlapping items has been documented. Meade and Gigone^16^ used overlapping and non-overlapping categorized wordlists as materials and measured the magnitude of collaborative inhibition in groups. Their experiments used three kinds of materials: the same exemplars for the same category, different exemplars from the same category, and different exemplars from a necessarily different category. Contrary to their hypotheses, collaborative inhibition was found regardless of item similarity. The researchers insisted that the retrieval-disruption hypothesis could partly explain their results. Specifically, for different items from the same category, retrieval disruption can occur if an item produced by one group member causes another member to retrieve an item from the same category but in contravention of the order of their retrieval strategy. For different items from different categories, retrieval disruption can result in category switching; for example, if a participant began by recalling from a non-overlapping category and then switched to recalling from an overlapping category. In line with this assumption, the manipulation of studying unrelated words should be less disruptive in collaboration, resulting in smaller or no collaborative inhibition effects when compared to the manipulation of studying categorized words. Although the magnitude of collaborative inhibition was smaller in our Experiment 1 (*M* = 0.06) compared to Meade and Gigone’s Experiment 1 (*M* = 0.11), this attenuated memory impairment still occurred in the current manipulation. These results indicated that even with the manipulation concerning minimizing word association and, thus, the lower chance of retrieval-disruption occurring during collaboration, collaborative inhibition still occurred. That is, other possible inducements should be explored.

In accordance with previous studies, we intended to maximally exclude the role of retrieval-disruption in the condition of non-overlapping items distributed among collaborative group members. Even with our mentioned manipulations, collaborative inhibition still occurred in the non-overlapping condition. What induced the collaborative inhibition when group members were instructed to recall totally non-overlapping items? Following these results obtained in Experiment 1, we further analysed the studies mentioned above concerning the effect of item similarity on collaborative inhibition. Therefore, we found one important aspect that might have induced the completely conflicting results: the instructions during the collaborative recall phase. In Basden *et al*.’s study^3^, participants were cautioned during the collaborative recall phase to refrain from helping or communicating with each other, and were strictly instructed to respond in turn. However, in another three studies^7,15,16^, collaborative group instructions were unstructured, so participants in collaborative groups mostly communicated with each other about recall items, judged the recall of others, and resolved disagreements between themselves. Could the different instruction manipulations have induced the different results, or does something else happen when participants recall information with different instructions?

The following experiment was guided by work on retrieval-induced forgetting^24^, which insists that when successfully completing a stem in the retrieval practice phase, participants inhibit their competing responses^24^. This inhibition should occur automatically if (a) a participant attempts to retrieve an item, and (b) competing responses are elicited in the process of retrieval. This assumption has also been shown to be true by collaborative memory research. Cuc *et al*.^25^ manipulated different instructions (accuracy or fluidity judgment to another’s recall) in the collaborative recall phase, which could induce different levels of response competition of recalled items that inhibit retrieval behaviour in turn, and found that different levels of response competition could influence the magnitude of the inhibition effect in collaboration. Considering the conclusions in studies on retrieval-induced forgetting and Cuc *et al*.’s study, although participants in the collaborative group were required to retrieve the studied items as much as possible in the above studies, the levels of response competition in the retrieval phase were not equal^3,7,15,16^. The strictly no-communication instruction eliminated the possible role of response competition^3^, while the similar unstructured instruction in Barber *et al*.’s^7^, Gummerum *et al*.’s^15^, Meade and Gigone’s studies^16^, was more likely to elicit the role of response competition in the retrieval phase.

To test this hypothesis, we then analysed the verbal coding results in our Experiment 1, which used an unstructured instruction in the collaboration but recall in turn manipulation. Tape recordings of each recall session were coded to determine the type of collaboration, if any, that occurred in the group in the overlapping or non-overlapping conditions. The coding scheme was based on Meade and Roediger^19^ and Meade and Gigone^16^. Specifically, for each word recalled, we coded whether the other members in each group responded with silence, or with a judgment upon the recalled word. Examples from the latter condition include statements such as ‘Have I studied that item?’ ‘That is a correct item’, ‘I don’t think we remember the same wordlist’, ‘Aha, we studied the same wordlist’, ‘I don’t think that item was in my study-list’, or ‘That was an incorrect word’. Examining whether group discussion occurred is important because one possible reason for the different results between Basden *et al*.’s study^3^ and the other three studies is that response competition may influence group recall performance. In our Experiment 1, instructions were unstructured in the collaborative recall phase, and all collaborative groups except one had a discussion during their collaboration. Hence, the collaborative inhibition we obtained here, which is inconsistent with Basden *et al*.^3^ and consistent with the aforementioned studies^7,15,16^, may have occurred because our manipulation elicited the role of response competition in the retrieval phase. In the following experiment, we further explored whether the collaborative inhibition effect occurs if there is low or no response competition.

Based on the above analyses and results, Experiment 2 contrasted four different conditions for retrieval which consisted of one nominal retrieval condition and another three collaborative retrieval conditions: (a) nominal recall: participants were encouraged to retrieve information individually, (b) low-response-competition collaborative recall: participants were encouraged to retrieve information in a group of two with the instruction that they recall in turn with strictly no communication, (c) medium-response-competition collaborative recall: participants were encouraged to retrieve information in a group of two with the instruction that they monitor their partner’s recall for superficial features, and (d) high-response-competition collaborative recall: participants were encouraged to retrieve information in a group of two with the instruction that they monitor their partner’s recall in regard to accuracy. These modes in Experiment 2 attempted to manipulate the different levels of response competition in the retrieval condition, as well as item similarity, and explore whether it could affect the magnitude of collaborative inhibition. If our results show that the magnitude of collaborative inhibition was equal among the different collaborative conditions, this should indicate that response competition was not the cause of the different results. If our results show that the magnitude of collaborative inhibition was not equal among the different collaborative conditions, this should indicate that response competition can influence collaborative inhibition. Furthermore, the amendment of retrieval-disruption or the possible role of other mechanisms in collaborative inhibition, aside from retrieval disruption, should be discussed.

## Experiment 2: Effects of response competition on collaborative inhibition

### Method

#### Participants

The participants in Experiment 2 were 84 undergraduates (mean age: 21.51 ± 0.70 years old) from Tianjin Normal University. The way the participants were assigned is shown in Table 2.

**Table 2:** Mean proportion of items’ recall errors as a function of the four different retrieval conditions in Experiment 2.

	Nominal recall	Collaborative recall		
		low-response -competition	medium-response -competition	high-response -competition
Number of groups	10	10	10	12
Proportion of recall errors	0.05 (0.06)	0.04 (0.04)	0.06 (0.04)	0.05 (0.04)

*Note*. Figures in parentheses denote standard deviations.

#### Design

Experiment 2 consisted of a 2 (item similarity: overlapping, non-overlapping) × 4 (retrieval condition: nominal recall, low-response-competition collaborative recall, medium-response-competition collaborative recall, high-response-competition collaborative recall) mixed-participants design. Item similarity was a within-participants variable. The retrieval condition was a between-participants variable. The nominal recall condition was manipulated so that participants recalled individually, while the last three retrieval conditions were manipulated as collaborative recall in which participants recalled in groups. These conditions were created to measure the effect of collaborative inhibition among different levels of response competition conditions. The primary dependent variable was the mean proportion of items correctly recalled in group- and individual-recall tasks.

#### Materials

Wordlists 1 and 2 from Experiment 1 were both randomly divided into halves and remixed with one half of wordlist 1 and one half of wordlist 2 to make two new wordlists for Experiment 2 (wordlists 4 and 5). Every half list was identical in regard to terms relating to affect, excitement, dominance, familiarity, strokes of the first character, and strokes of the last character. In the new wordlists, there were both 15 overlapping items and 15 non-overlapping items (i.e. wordlist 1 A + wordlist 2 A = wordlist 4; wordlist 1 A + wordlist 2B = wordlist 5). Both were 30-word lists (10 verbs, 10 nouns, and 10 adjectives) that were identical in regard to terms relating to affect, excitement, dominance, familiarity, strokes of the first character, and strokes of the last character. The words used in Experiment 2 are also listed in the Supplementary material in Chinese, along with their English translations.

#### Procedure

The procedure for Experiment 2 was identical to that of Experiment 1, with three exceptions. First, as in Experiment 1, item similarity was manipulated as a within-participants design. However, different from Experiment 1, wherein items were presented in blocks, in Experiment 2 the overlapping and non-overlapping items were presented in a mixed manner. A mixed-design is more appropriate for participants in collaborative groups to monitor whether they had been given the same wordlist to learn (high-response-competition collaborative recall condition). Second, before the formal experiments, participants had a practice phase with different, shorter wordlists (a six-word list). The practice phase was a shorter version of the formal experiments to ensure all participants were clearly aware of the different collaborative instructions. Once they had correctly finished the practice phase, the formal experiment began. Finally, for Experiment 2, we conducted four retrieval conditions in the recall session. Participants in the nominal recall condition were instructed to recall individually. Participants in the low-response-competition collaborative recall condition were instructed to recall in turn in a collaborative group, with strictly no other communication involved, which manipulation was similar to that in Basden *et al*.’s Experiment 3. Participants in the medium-response-competition collaborative recall condition were instructed to recall by turn-taking and making superficial judgments for each result recalled by others (i.e. checking whether the strokes of a character were correct in Chinese; this is analogous to checking whether they were spelt correctly, as in non-Chinese languages such as English). Finally, participants in the high-response-competition collaborative recall condition were instructed to recall by turn-taking and judging if the items recalled by others were correct items in regard to their *own* study list. The latter two retrieval conditions were manipulated similar to that in the current Experiment 1.

## Results

### Group recall performance

To compare the current results with our Experiment 1, we first analysed the magnitude of collaborative inhibition in overlapping and non-overlapping item conditions, by collapsing the three collaborative retrieval conditions and compared their retrieval performance with that of the nominal retrieval condition. A 2 (item similarity: overlapping, non-overlapping) × 2 (retrieval condition: nominal, collaborative) mixed-factor ANOVA on correct recall showed that the main effect of item similarity was statistically significant, *F* (1, 40) = 111.30, *p < *0.001, partial η^2^ = 0.74. The main effect of retrieval condition was statistically significant, *F* (1, 40) = 7.86, *p* = 0.008, partial η^2^  = 0.16, which means collaborative groups (collapsed across response competition levels) recalled less than their nominal peers. What is more important, there was no interaction between item similarity and retrieval condition, *F* (1, 40) = 0.01, *p* = 0.93, which consolidated the results obtained in our Experiment 1.

Subsequently, we further analysed the magnitude of collaborative inhibition among different levels of response competition conditions. A 2 (item similarity: overlapping, non-overlapping) × 4 (retrieval condition: nominal recall, low-response-competition collaborative recall, medium-response-competition collaborative recall, high-response-competition collaborative recall) mixed-factor ANOVA on correct recall showed that the main effect of item similarity was significant, *F* (1, 38) = 146.81, *p < *0.001, partial η^2^  = 0.79. The main effect of retrieval condition was significant, *F* (3, 38) = 3.93, *p* = 0.015, partial η^2^ = 0.24. The primary aim of this experiment was to answer the question: Does the magnitude of collaborative inhibition depend upon the different levels of response competition in a group? Thus, we carried out a one-way analysis of variance (ANOVA) with post hoc comparisons, which involved Bonferroni corrections, that showed that the recall performance of nominal-group members was significantly better than collaborative group members in the medium-response-competition condition (*p* = 0.036) and the collaborative-group members in the high-response-competition condition (*p* = 0.040). This indicated that, in these two retrieval conditions, collaborative-group members showed the classical collaborative inhibition effect. However, there was no significant difference in recall performance between nominal-group members and the collaborative-group members in the low-response-competition condition (*p* > 0.05). In addition, the interaction between item similarity and retrieval condition was non-significant, *F* (3, 38) = 0.36, *p* = 0.78.

After analysing the ANOVAs above, a Levene’s test on correct recall in groups showed that deviations in the overlapping condition were symmetrical, *F* (3, 38) = 0.408, *p* = 0.748, which results could be considered as normally distributed (Skewness = 0.228, Kurtosis = −0.488); and deviations in the non-overlapping condition were symmetrical, *F* (3, 38) = 0.305, *p* = 0.822, which results could also be considered as normally distributed (Skewness = 0.297, Kurtosis = 0.642).

### Group recall errors

As in Experiment 1, we calculated the recall errors among conditions. However, different from Experiment 1, wherein items were presented in blocks, the overlapping and non-overlapping items were presented in a mixed manner in Experiment 2, which means participants recalled both kinds of items in one answer sheet. We respectively calculated the proportion correctly recalled in overlapping and non-overlapping conditions in a group; however, recall errors could not easily be classified as overlapping or non-overlapping items in a mixed presentation manner. Thus, the recall errors reported here were collapsed into item similarity variables, which are shown in Table 2. A one-way ANOVA on recall errors showed that there was no main effect of retrieval condition, *F* (3, 38) = 0.31, *p* = 0.82. This finding is consistent with previous research, suggesting that when a collaborative group recalls in turn, no error-pruning effect is present in the collaboration^26^.

A Levene’s test on recall errors in groups showed that deviations in the overlapping condition were symmetrical, *F* (3, 38) = 0.054, *p* = 0.983, while the errors in the overlapping condition were somewhat non-normally distributed (Skewness = 1.425, Kurtosis = 2.301); deviations in the non-overlapping condition were symmetrical, *F* (3, 38) = 0.118, *p* = 0.949, which results could be considered as normally distributed (Skewness = 0.857, Kurtosis = −0.211). As in Experiment 1, we also conducted a Friedman test on recall errors between overlapping and non-overlapping conditions, and the results showed that there was a statistically significant difference between groups, *χ* ^2^ = 30.118, *p* < 0.001, which was consistent with the parametric testing results. As a result, the parametric testing used in Experiment 2 was considered to be valid.

### Subsequent individual recall performance

The primary purpose of the current experiment was to detect the influence of response competition on collaborative inhibition. We also calculated the subsequent individual recall performance to test how correct recall changed from collaborative recall to subsequent individual recall as a function of previous response competition. In Experiment 2, we collapsed the overlapping and non-overlapping conditions’ subsequent individual recall performance because it was not the major concern of the current experiment.

Therefore, we conducted one-way ANOVA on the correct recall in the subsequent individual recall test among the nominal group members, the low-response-competition collaborative group members, the medium-response-competition collaborative group members, and the high-response-competition collaborative group members. The results showed that there was no main effect among conditions, *F* (3, 80) = 0.56, *p* = 0.64. This was consistent with the results obtained in Experiment 1, which indicated that the collaborative inhibition effect disappeared in the subsequent individual recall task^11^.

A Levene’s test on correct recall in subsequent individual recall showed that the deviations among conditions were symmetrical, *F* (3, 80) = 1.910, *p* = 0.135, which results could be considered as normally distributed (Skewness = 0.226, Kurtosis = −0.293).

## Discussion

The results of Experiment 2 demonstrated the role of response competition, which might have influenced the effect of collaborative inhibition. Regarding previous research on collaboration, Cuc *et al*.^25^ used two levels of response competition in collaborative groups to detect the effect on a post-collaborative recall test. The results showed that participants in the accuracy monitoring (high-response-competition) condition showed classical collaborative inhibition while participants in the superficial monitoring (medium- or low-response-competition) condition did not, which means the manipulation of response competition could influence the magnitude of the collaborative inhibition effect. Several psychologists supported Cuc *et al*.’s conclusion, which suggests that collaborative inhibition is a result of the inhibitory processes that function to resolve interference during retrieval, and attributed the forgetting to the suppression of an item’s representation^7,25,27,28^. Our manipulation of retrieval conditions elicited competing responses in the medium- and high-response-competition conditions compared to the low-response-competition condition. The results in our Experiment 2 showed that collaborative inhibition occurred in both overlapping and non-overlapping conditions, which consolidated our findings in Experiment 1. What is more important, the results showed that collaborating group members in medium- and high-response-competition conditions exhibited the classical collaborative inhibition effect, whereas no such effect was shown in the low-response-competition condition. These results indicated that other factors, such as retrieval inhibition, also contribute to collaborative inhibition, which is consistent with retrieval-induced forgetting studies^24,29^.

Interestingly, however, our results showed that collaborative group members in the medium- and high-response-competition conditions demonstrated an equal collaborative inhibition effect, which was different from Cuc *et al*.’s study^25^. The different results might be due to the different characteristics between English words and Chinese characters that were presented as studied materials. Chinese characters are a type of ideogram and, because of their visual complexity, have significant differences compared to alphabetical material. In addition, Chinese characters can be accessed directly by the activation of graphic information, which also distinguishes them from alphabetical materials^30^. In our Experiment 2, participants in the medium-response-competition collaborative recall condition experienced the same character representation as in the high-response-competition collaborative recall condition. Thus, the levels of response competition were the same for the medium- and high-response competition conditions in the collaboration and, consequently, the magnitude of collaborative inhibition in these two retrieval conditions was the same. Contrary to the above two conditions, participants in the low-response-competition collaborative recall condition exhibited no collaborative inhibition effect. The manipulation of the low-response-competition collaborative recall condition in Experiment 2 was similar to that in Basden *et al*.’s study^3^, which possibly excluded not only the role of retrieval-disruption, but also the role of response competition in collaborative recall, and as a result, the effect of collaborative inhibition disappeared.

## General Discussion

In our two experiments, we systematically explored possible reasons for the conflicting results among the aforementioned studies^3,7,14–16^. Moreover, we examined the core viewpoint of the retrieval-disruption hypothesis to test if it could remain as the sole mechanism underlying collaborative inhibition. Experiment 1 closely followed the methods of the relevant studies^3,15,16^, employing overlapping and non-overlapping unrelated words as studied materials, and the related instructions for collaboration were unstructured. Based on the results from Experiment 1, we further explored another candidate, response competition. This was conducted by manipulating four retrieval conditions during recall to detect the possible influence of response competition on collaborative inhibition. The results of our study provide evidence, in regard to a Chinese cultural context, of other factors, or even multiple mechanisms, as suggested by Barber *et al*.^7^, underlying collaborative inhibition besides retrieval disruption.

Previous studies concerning the effect of item similarity on collaborative inhibition have obtained conflicting results. As we mentioned in the introduction section, only two studies have concluded that collaborative inhibition disappeared when they manipulated the item similarity variable^3,14^, which is inconsistent with other results^7,15,16^. After thoroughly analysing the results of the former two studies, we found that the interaction of retrieval condition (collaborative or nominal) and item similarity (overlapping or non-overlapping) was non-significant (*p* = 0.11) in Basden *et al*.’s Experiment 3, but they still conducted a simple test to conclude that collaborative inhibition disappeared when participants were required to recall different categories in a group^3^. Based on these results, they suggested that the retrieval-disruption mechanism could completely predict collaborative inhibition by manipulating item similarity variables. Similarly, the interaction of the same two variables was also marginally significant (*p* = 0.09) in Barber *et al*.’s study^14^, and they conducted a series of independent *t* tests after ANOVA to conclude that collaborative inhibition was statistically absent when information was non-overlapping in a collaborative group. Could these statistically non-significant results accurately support the core viewpoint of the retrieval-disruption hypothesis as the authors insisted? In another two studies mentioned above, the results consistently showed that there was no statistically significant interaction between retrieval condition and item similarity (*p* > 0.05), which indicated that collaborative inhibition persisted no matter whether participants were required to recall the same or different items in a group^15,16^. Along with these results, they suggested that in conditions that maximally eliminate the influence of retrieval-disruption in collaboration, there might exist other mechanisms that induce collaborative inhibition^15,16^. After a comprehensive analysis of the aforementioned studies’ results, we hypothesized that retrieval-disruption has little or no influence in the non-overlapping recalled items collaborative groups. Based on the analyses, in the current study, we first explored the controversial issue that is focused on the influence of item similarity on collaborative inhibition, and the results showed that there was no significant interaction between item similarity and retrieval condition variables (*p* = 0.33 in Experiment 1, and *p* = 0.93 in Experiment 2), which indicated that collaborative inhibition occurred regardless of the similarity of recalled items in a group. However, the current sample sizes were somewhat smaller than those in previous studies (which were actually 28 dyads in Experiment 1 and 42 dyads in Experiment 2), which indicate that our study was somewhat underpowered to observe small effects. To be clear, the purpose of the current study was not to totally deny the influence of retrieval-disruption in the non-overlapping recalled items condition, but rather to explore whether there were other factors that could also influence collaborative inhibition in some specific conditions besides retrieval-disruption.

Based on the verbal coding results in our Experiment 1, combined with the analyses of previous studies, we manipulated the response competition variable in group recall, in addition to item similarity, to detect its possible influence on collaborative inhibition. The results showed that collaborative inhibition disappeared in the low-response-competition collaborative recall condition, from which we concluded that the levels of response competition in a group could affect the magnitude of collaborative inhibition, especially in the non-overlapping recalled items condition. Individual studies have already demonstrated that response competition of the given items could influence the magnitude of the inhibition effect of the non-given items, and, what is more important, Cuc *et al*.^25^ also demonstrated that the higher level of response competition in a collaborative group produces a greater collaborative inhibition effect. Our results in Experiment 2 further confirm the role of response competition in collaborative inhibition apart from retrieval-disruption.

However, contrary to our results obtained in Experiment 2, previous studies demonstrated the equal magnitude of collaborative inhibition in a turn-taking and a free-flowing collaborative procedure^21–23^. Thorley and Dewhurst observed classical collaborative inhibition in a strictly turn-taking collaborative recall condition, and the magnitude of this effect was equal to that in a free-for-all method collaborative recall condition^22^. Another study also found equivalent collaborative inhibition between a forced consensus paradigm and a strictly turn-taking procedure^23^. The possible reasons underlying the conflicting results between the current and previous two studies might be as follows: on the one hand, the research aim of the current study was to detect whether there were other cognitive factors that could influence collaborative inhibition by maximally eliminating the role of retrieval-disruption. Thus, in the condition of low-response-competition collaborative recall, we eliminated not only the role of retrieval-disruption, but also the role of retrieval-inhibition in collaboration. The results showed that collaborative inhibition disappeared in the low-response-competition collaborative recall condition. However, in the study mentioned above, Thorley and Dewhurst used 20 wordlists that were found to produce the highest incidence of false recall as studied materials, in accordance with their research aims, and found an equal magnitude of collaborative inhibition in a turn-taking and a free-flowing collaborative procedure. The results observed in that study might have occurred because of the stable influence of retrieval-disruption in the overlapping information distributed in collaborative group members^22^. Similar results were obtained when participants were required to recall personal, shared memories by using an autobiographical interview^23^; the researchers insisted that social factors also contributed to collaborative recall performance besides retrieval-disruption. That is, unlike the previous two studies, our Experiment 2 strictly manipulated collaborative procedure by maximally excluding retrieval-disruption and other social factors to detect the role of retrieval-inhibition in collaborative inhibition. The results we obtained here more strictly demonstrated the role of monitoring conditions on collaborative inhibition, but not other factors involved in a free-for-all method. On the other hand, in accordance with their specific research aims, previous studies that demonstrated equivalent collaborative inhibition between a free-for-all paradigm and a turn-taking procedure only focused on the overlapping condition^22,23^, which means their conclusions concerning the effect of different recall procedures on collaborative inhibition cannot extend to the non-overlapping condition. The results in Experiment 2 demonstrated that response competition could affect collaborative memory regardless of item similarity, which results could be explained by the retrieval-inhibition hypothesis of collaborative inhibition^25^.

The current study did not confirm the small effect of retrieval-disruption underlying collaborative inhibition in the non-overlapping items condition reported in Basden *et al*.’s Experiment 3. Two possible reasons should be discussed: First, retrieval-disruption mainly induced the collaborative inhibition effect among conditions. Thus, according to our research aims, we maximally eliminated the role of retrieval-disruption by manipulating the item similarity variable and the effect of collaborative inhibition was attenuated. Second, considering the results reported in our Experiment 2, it could be that the elimination of response competition in Basden *et al*.’s Experiment 3 but not the elimination of retrieval-disruption in collaboration in the non-overlapping condition, caused the effect of collaborative inhibition to disappear. These results supported the role of the retrieval-inhibition explanation underlying collaborative inhibition. Our Experiment 2 tried to link the individual memory conclusions to collaborative memory, and the results showed that collaborative group members in high- and medium-response-competition collaborative recall conditions showed the classical collaborative inhibition effect, whereas no such effect was shown in the low-response-competition condition. The results presented here could not be explained by the retrieval-disruption hypothesis for participants in different collaborative retrieval conditions experiencing a similar extent of retrieval-disruption from their original information organization. The results of Experiment 2 consolidated our findings in Experiment 1, and further supported the explanation of the retrieval inhibition hypothesis, which states that response competition affects memory performance.

Another possible mechanism that might have induced collaborative inhibition in the non-overlapping item condition in the current study is the ‘group interaction’ itself, which has not previously been shown to be involved in collaborative inhibition^31^. However, several studies have supported the contribution of group interaction to collaborative memory^16,19,32^. Meade and Gigone^16^ used verbal coding methodology to analyse the social-process variables (i.e. acknowledgements) in an unstructured instruction condition, and concluded that group interaction contributed to collaborative inhibition. On the contrary, the strictly no-communication and recall-in-turn instruction in Basden *et al*.^3^ completely eliminated possible interaction. This occurred through removing communication or limiting the initiative of group members and forcing them to take ordered rather than spontaneous turns contributing items to the group product. The instruction in Basden *et al*.’s study^3^ not only eliminated the role of response competition in a group, but also eliminated any possible role of group interaction in collaboration, which has been demonstrated to influence groups’ production^19,32^. The result of the recall performance in the low-response-competition collaborative recall condition in our Experiment 2 indicated that such instruction could eliminate the robust effect of collaborative inhibition, which is consistent with Basden *et al*.’s Experiment 3^3^. The role of such group interaction, which requires further exploration in future, can be considered in conjunction with cognitive explanations.

As mentioned before, our research aim was not to question but to amend the role of retrieval-disruption in collaborative inhibition. In our two experiments, we observed that the collaborative inhibition effect disappeared in a subsequent individual recall task, which was consistent with the retrieval-disruption explanation^3,11^. However, these results do not detract from our purpose, which is to explore retrieval disruption and determine whether other factors exist that can affect collaborative memory. The analysis of subsequent individual recall tasks might further support the theory that multiple mechanisms underlie collaborative inhibition, as suggested by Barber *et al*.^7^.

There are limitations to the generalizability of the results because of the small sample size in the current study. As mentioned earlier, previous studies provided evidence for a small effect of retrieval-disruption in the non-overlapping condition. However, due to the limitation of the sample size in our study, it was somewhat underpowered to observe a small effect. Therefore, a large sample size would be more appropriate for future studies. Limitations may also arise because of the between-participants design of the retrieval conditions variable in the current two experiments. Although previous studies demonstrated that this manipulation was acceptable to measure the collaborative inhibition effect, it is better to test the effect using a within-participants design or require all participants undergo individual recall tests before group recall, to maximally eliminate differences in recall performance between groups in future studies.

The goal of the current study was to discern a possible reason for the differences in the results of previous studies, and to tentatively investigate possible mechanisms underlying collaborative inhibition. Significant collaborative inhibition occurred for both overlapping and non-overlapping conditions in Experiment 1. The magnitude of collaborative inhibition was influenced by response competition in Experiment 2. Considered together, the results suggest that besides retrieval-disruption, other factors (i.e. retrieval-inhibition) also contribute to collaborative inhibition.

## Electronic supplementary material

Supplementary Material
