# Supplementary material for: The Effect of Item Similarity and Response Competition Manipulations on Collaborative Inhibition in Group Recall
Source: Sci Rep. 2017 Sep 20;7:11946. doi: 10.1038/s41598-017-12177-x (PMC5607282; doi:10.1038/s41598-017-12177-x)
Supplement: Supplementary file 1 — Supplementary Material [file 41598_2017_12177_MOESM1_ESM.pdf]

The Effect of Item Similarity and Response Competition Manipulations on Collaborative Inhibition in Group Recall

Huan Zhang[1, 2], Yao Fu[1, 3], Xingli Zhang[1*], and Jiannong Shi[1, 4]

[1]CAS Key Laboratory of Behavioural Science, Institute of Psychology, Beijing, China

[2]Department of Psychology, School of Education Science, Tianjin Normal University, Tianjin, China

[3]University of Chinese Academy of Sciences, Beijing, China

[4]Department of Learning and Philosophy, Aalborg University, Aalborg, Denmark

[*]Correspondence concerning this article should be addressed to
Xingli Zhang (Room 519, Courtyard 16, Lincui Road, Chaoyang District, Beijing, China; Email: zhangxl@psych.ac.cn)

Supplementary material:

Experiment 1*

World List 1

No.	Chinese Version	English Transation	Affect		Excitement		Dominance		Familarity		strokes in the first character	strokes in the last character
			M	*SD*	*M*	*SD*	*M*	*SD*	*M*	*SD*		
1	辩护	defend	5.20	1.32	5.23	1.68	5.42	1.64	4.60	2.07	16.00	7.00
2	督促	urger	4.76	1.61	4.47	1.74	5.33	1.80	5.10	1.86	13.00	9.00
3	观望	hesitate	4.91	1.62	4.08	1.57	5.94	1.53	4.88	1.85	6.00	11.00
4	减肥	reduce-weight	4.99	1.68	4.62	2.21	6.15	1.87	5.79	2.21	11.00	8.00
5	评估	assess	4.99	1.41	3.89	1.55	5.03	1.76	5.00	2.01	7.00	7.00
6	请求	request	5.01	1.68	4.60	1.92	5.79	1.82	5.98	1.83	10.00	7.00
7	侍候	look-after	4.89	1.88	4.52	3.00	5.94	1.75	4.81	2.06	8.00	10.00
8	调节	adjust	5.09	1.34	3.65	1.46	5.49	1.87	5.35	2.22	10.00	5.00
9	突围	break-out	4.97	1.68	5.48	2.07	5.02	1.87	4.30	2.11	9.00	7.00
10	追逐	pursue	5.03	1.51	5.14	1.74	5.92	1.81	5.46	1.84	9.00	10.00
11	帮主	chief	5.09	2.12	4.73	2.06	3.79	1.74	3.45	2.16	9.00	5.00
12	法制	legality	5.00	1.54	4.68	1.77	3.01	1.50	5.31	2.04	8.00	8.00
13	皇帝	emperor	5.17	1.64	5.34	2.20	2.70	1.79	3.81	2.33	9.00	9.00
14	军队	army	4.97	1.67	5.69	1.90	2.92	1.50	4.59	2.16	6.00	4.00
15	脸色	complexion	4.78	1.64	4.42	2.01	6.07	1.65	6.07	1.82	11.00	6.00
16	命运	destiny	5.12	1.78	5.48	2.17	4.65	2.51	5.50	1.84	8.00	7.00
17	狮子	lion	5.10	1.58	5.56	2.36	3.08	1.61	4.88	2.31	9.00	3.00
18	铁路	railway	5.10	1.15	3.43	1.93	4.38	2.01	5.36	2.24	10.00	13.00
19	性命	life	4.91	1.79	5.93	2.37	4.59	2.14	5.47	2.04	8.00	8.00
20	政府	government	4.85	1.39	4.46	2.08	2.85	1.75	5.13	2.11	9.00	8.00
21	彻底	thoroughly	5.38	1.51	4.43	1.90	5.01	1.70	5.18	2.03	7.00	8.00
22	纯粹	purely	5.21	1.30	4.05	1.71	5.37	1.73	4.62	1.80	7.00	14.00
23	粗壮	stockily	4.95	1.46	4.52	1.81	5.21	1.71	5.20	1.91	11.00	6.00
24	骄傲	proudly	4.94	1.93	4.86	1.95	6.00	1.55	5.72	1.83	9.00	12.00
25	离奇	quaintly	4.79	1.76	6.05	1.90	4.55	1.81	4.21	1.68	10.00	8.00
26	忙碌	busily	4.95	1.49	4.47	1.74	5.86	1.69	5.89	1.90	6.00	13.00
27	奇怪	strangely	5.08	1.53	5.61	1.70	4.77	1.77	5.64	1.94	8.00	8.00
28	柔弱	feebly	4.58	1.64	3.91	1.66	6.05	1.62	4.90	1.89	9.00	10.00
29	淘气	naughtily	5.37	1.55	4.70	1.87	6.08	1.68	5.82	1.73	11.00	4.00
30	依旧	still	5.03	1.46	3.45	1.72	5.10	1.70	5.08	2.03	8.00	5.00

World List 2

No.	Chinese Version	English Transation	Affect		Excitement		Dominance		Familarity		strokes in the first character	strokes in the last character
			M	*SD*	*M*	*SD*	*M*	*SD*	*M*	*SD*		
1	阐述	elaborate	5.06	1.18	3.57	1.61	6.11	1.44	4.87	1.92	11.00	8.00
2	反击	beat-back	4.87	1.79	5.72	1.90	5.34	1.93	4.70	2.16	4.00	5.00
3	害羞	shy	4.93	1.63	4.13	1.91	5.75	1.86	5.58	1.81	10.00	10.00
4	解除	relieve	4.87	1.57	4.66	1.73	5.62	1.72	4.90	2.01	13.00	9.00
5	强调	emphasize	4.90	1.58	5.18	1.97	5.59	1.71	5.75	1.94	12.00	10.00

6	认错	apology	5.09	1.61	4.78	1.87	5.83	1.94	5.70	1.91	4.00	13.00
7	搜寻	seek	4.94	1.66	5.06	1.90	5.65	1.67	5.28	1.99	12.00	6.00
8	同情	sympathize	5.22	1.66	4.09	1.85	6.25	1.65	5.95	1.93	6.00	11.00
9	挖掘	dig	5.14	1.63	4.78	1.78	5.44	1.64	4.53	1.87	9.00	11.00
10	主宰	decide	4.74	2.02	4.88	1.95	4.51	2.00	4.39	1.87	5.00	10.00
11	边境	border	4.90	1.17	4.32	1.85	4.04	1.90	4.08	1.84	5.00	14.00
12	阁下	monseigneur	5.16	1.64	3.54	1.87	4.11	1.98	3.55	2.07	9.00	3.00
13	纪律	discipline	5.12	1.61	4.24	1.77	3.93	1.98	5.73	1.95	6.00	9.00
14	开支	expenses	5.11	1.57	4.55	2.00	6.03	1.73	5.37	2.04	4.00	4.00
15	烈士	martyr	5.18	2.20	5.91	2.02	3.50	1.82	4.18	2.12	10.00	3.00
16	农村	village	4.99	1.63	3.89	1.88	5.41	1.97	5.99	2.00	6.00	7.00
17	事物	object	5.17	1.27	3.01	1.71	5.55	1.86	5.63	2.13	8.00	8.00
18	消防	fire-fighting	5.06	1.63	4.59	1.81	4.57	1.85	4.94	1.86	10.00	6.00
19	遗产	legacy	5.26	1.91	5.74	2.18	4.85	2.32	4.34	2.06	12.00	6.00
20	职责	duty	5.27	1.55	4.84	1.92	4.99	1.83	5.35	1.82	11.00	8.00
21	沉醉	immersed	4.87	1.96	4.42	2.01	5.63	1.80	5.22	1.68	7.00	15.00
22	匆忙	hastily	4.70	1.70	4.63	1.86	5.76	1.77	5.85	2.07	5.00	6.00
23	坚硬	hardy	5.24	1.59	4.38	2.03	4.98	1.90	5.66	1.94	7.00	12.00
24	考究	well-dressed	5.38	1.65	4.34	2.00	5.28	1.83	4.68	2.07	6.00	7.00
25	厉害	badly	4.90	1.78	5.54	1.89	5.08	1.89	5.53	1.93	5.00	10.00
26	内向	bashfully	4.66	1.44	3.61	1.62	5.40	1.83	6.03	1.67	4.00	6.00
27	谦卑	humbly	4.99	1.84	4.24	1.88	5.40	1.93	5.03	1.89	12.00	8.00
28	深入	deeply	5.44	1.47	4.19	1.72	5.66	1.41	4.89	2.18	11.00	2.00
29	威严	imperially	5.20	1.74	5.17	1.98	3.98	1.91	4.74	1.85	9.00	7.00
30	早熟	prematurely	5.06	1.38	4.81	1.96	5.15	1.74	5.55	1.78	6.00	15.00

World List 3

No.	Chinese Version	English Transation	Affect		Excitement		Dominance		Familarity		strokes in the first character	strokes in the last character
			M	*SD*	*M*	*SD*	*M*	*SD*	*M*	*SD*		
1	吃惊	surprise	4.82	1.71	5.54	2.13	5.40	1.62	5.52	2.04	6.00	11.00
2	覆盖	cover	5.18	1.49	3.92	1.51	5.35	1.79	5.13	1.94	18.00	11.00
3	呼吁	appeal	5.07	1.68	4.93	1.97	5.86	1.66	5.32	1.77	8.00	6.00
4	履行	perform	5.12	1.64	4.40	1.82	5.82	1.81	4.90	2.01	15.00	6.00
5	抢险	rescue	4.90	1.84	5.92	1.84	4.96	1.79	5.10	2.13	7.00	9.00
6	散发	emit	5.14	1.44	3.87	1.47	5.32	1.61	4.86	1.74	12.00	5.00
7	填补	fill	4.96	1.49	3.49	1.70	5.49	1.62	4.64	1.81	13.00	7.00
8	透露	reveal	4.85	1.54	4.44	1.82	5.98	1.45	5.15	1.95	10.00	21.00
9	整顿	reorganize	5.19	1.39	4.38	2.05	5.20	1.77	5.29	5.00	16.00	10.00
10	自卫	defend	4.89	1.65	5.47	2.02	6.29	1.81	4.88	1.71	6.00	3.00
11	对策	treatment	5.14	1.47	4.46	1.72	5.56	1.65	5.14	1.95	5.00	12.00
12	宫廷	palace	5.03	1.97	4.77	2.16	3.19	1.94	3.83	2.25	9.00	6.00
13	警官	police	4.83	1.53	4.79	1.96	3.25	1.67	4.75	2.11	19.00	8.00
14	廉价	cheap	4.87	1.88	5.94	1.86	5.29	1.86	5.70	1.90	13.00	6.00
15	秘书	secretary	5.23	1.42	3.58	1.88	5.77	1.78	4.73	2.17	10.00	4.00
16	契约	contract	5.05	1.58	4.54	1.78	5.05	2.03	4.57	2.03	9.00	6.00

17	谈判	negotiation	4.72	1.29	5.20	1.94	5.47	1.72	4.51	2.05	10.00	7.00
18	心脏	heart	5.05	1.23	4.33	1.91	4.81	2.16	5.67	1.85	4.00	10.00
19	医生	doctor	5.18	1.68	4.12	1.84	3.85	1.58	5.56	2.08	7.00	5.00
20	总管	manager	4.70	1.49	4.05	1.61	3.67	1.84	4.08	2.06	9.00	14.00
21	初步	preliminary	4.85	1.18	3.26	1.46	6.02	1.36	5.03	1.90	7.00	7.00
22	凑巧	luckily	5.06	1.64	4.52	1.74	4.60	1.90	5.11	2.12	11.00	5.00
23	简易	easily	5.16	1.61	3.74	1.71	6.07	1.57	4.87	2.14	13.00	8.00
24	夸张	dramatically	4.76	1.74	5.07	1.79	5.88	1.50	5.52	1.91	6.00	7.00
25	零星	sporadically	4.64	1.36	3.17	1.47	5.57	2.02	4.59	1.89	13.00	9.00
26	平滑	smoothly	5.06	1.56	3.43	1.41	5.60	1.51	4.81	2.03	5.00	12.00
27	日常	daily	5.20	1.15	3.40	1.67	5.74	1.68	5.87	2.20	4.00	11.00
28	适度	moderately	5.33	1.22	3.73	1.80	5.84	1.74	5.37	2.01	9.00	9.00
29	稀罕	rare	5.38	1.69	5.33	1.94	4.59	1.92	4.79	1.98	12.00	7.00
30	炽热	baking	5.43	1.61	5.03	2.02	5.13	1.82	4.95	2.01	9.00	10.00

*We selected 90 unrelated neutral words from the Chinese Affective Words System: 30 verbs, 30 nouns, and 30 adjectives. Affective differences were not significant among the three kinds of words: Mverbs = 4.99, SDverbs = 0.13; Mnouns = 5.04, SDnouns = 0.16; Madjectives = 5.05, SDadjectives = 0.25, $F(2, 87) = 0.89$, $p > 0.05$. In Experiment 1, 90 words were put into three 30-word lists (10 verbs, 10 nouns, and 10 adjectives) that were matched in affect (M1 = 5.01, SD1 = 0.17; M2 = 5.05, SD2 = 0.19; M3 = 5.03, SD3 = 0.20; $F(2, 87) = 0.34$, $p > 0.05$), excitement (M1 = 4.72, SD1 = 0.71; M2 = 4.56, SD2 = 0.68; M3 = 4.43, SD3 = 0.73; $F(2, 87) = 1.17$, $p > 0.05$), dominance (M1 = 4.94, SD1 = 1.09; M2 = 5.18, SD2 = 0.71; M3 = 5.22, SD3 = 0.81; $F(2, 87) = 0.91$, $p > 0.05$), familiarity (M1 = 5.10, SD1 = 0.63; M2 = 5.13, SD2 = 0.64; M3 = 5.01, SD3 = 0.46; $F(2, 87) = 0.38$, $p > 0.05$), strokes in the first character (M1 = 9.01, SD1 = 2.12; M2 = 7.97; SD2 = 2.95, M3 = 9.83, SD3 = 3.97; $F(2, 87) = 2.74$, $p > 0.05$), and strokes in the last character (M1 = 8.00, SD1 = 2.77; M2 = 8.30, SD2 = 3.44; M3 = 8.40, SD3 = 3.56; $F(2, 87) = 0.12$, $p > 0.05$).

Experiment 2*

World List 4

No.	Chinese Version	English Transation	Affect		Excitement		Dominance		Familarity		strokes in the first character	strokes in the last character
			M	*SD*	*M*	*SD*	*M*	*SD*	*M*	*SD*		
1	辩护	defend	5.20	1.32	5.23	1.68	5.42	1.64	4.60	2.07	16.00	7.00
2	督促	urger	4.76	1.61	4.47	1.74	5.33	1.80	5.10	1.86	13.00	9.00
3	观望	hesitate	4.91	1.62	4.08	1.57	5.94	1.53	4.88	1.85	6.00	11.00
4	减肥	reduce-weight	4.99	1.68	4.62	2.21	6.15	1.87	5.79	2.21	11.00	8.00
5	评估	assess	4.99	1.41	3.89	1.55	5.03	1.76	5.00	2.01	7.00	7.00
6	帮主	chief	5.09	2.12	4.73	2.06	3.79	1.74	3.45	2.16	9.00	5.00
7	法制	legality	5.00	1.54	4.68	1.77	3.01	1.50	5.31	2.04	8.00	8.00
8	皇帝	emperor	5.17	1.64	5.34	2.20	2.70	1.79	3.81	2.33	9.00	9.00
9	军队	army	4.97	1.67	5.69	1.90	2.92	1.50	4.59	2.16	6.00	4.00
10	脸色	complexion	4.78	1.64	4.42	2.01	6.07	1.65	6.07	1.82	11.00	6.00
11	彻底	thoroughly	5.38	1.51	4.43	1.90	5.01	1.70	5.18	2.03	7.00	8.00
12	纯粹	purely	5.21	1.30	4.05	1.71	5.37	1.73	4.62	1.80	7.00	14.00
13	粗壮	stockily	4.95	1.46	4.52	1.81	5.21	1.71	5.20	1.91	11.00	6.00
14	骄傲	proudly	4.94	1.93	4.86	1.95	6.00	1.55	5.72	1.83	9.00	12.00
15	离奇	quaintly	4.79	1.76	6.05	1.90	4.55	1.81	4.21	1.68	10.00	8.00
16	阐述	elaborate	5.06	1.18	3.57	1.61	6.11	1.44	4.87	1.92	11.00	8.00
17	反击	beat-back	4.87	1.79	5.72	1.90	5.34	1.93	4.70	2.16	4.00	5.00
18	害羞	shy	4.93	1.63	4.13	1.91	5.75	1.86	5.58	1.81	10.00	10.00
19	解除	relieve	4.87	1.57	4.66	1.73	5.62	1.72	4.90	2.01	13.00	9.00

20	强调	emphasize	4.90	1.58	5.18	1.97	5.59	1.71	5.75	1.94	12.00	10.00
21	边境	border	4.90	1.17	4.32	1.85	4.04	1.90	4.08	1.84	5.00	14.00
22	阁下	monseigneur	5.16	1.64	3.54	1.87	4.11	1.98	3.55	2.07	9.00	3.00
23	纪律	discipline	5.12	1.61	4.24	1.77	3.93	1.98	5.73	1.95	6.00	9.00
24	开支	expenses	5.11	1.57	4.55	2.00	6.03	1.73	5.37	2.04	4.00	4.00
25	烈士	martyr	5.18	2.20	5.91	2.02	3.50	1.82	4.18	2.12	10.00	3.00
26	沉醉	immersed	4.87	1.96	4.42	2.01	5.63	1.80	5.22	1.68	7.00	15.00
27	匆忙	hastily	4.70	1.70	4.63	1.86	5.76	1.77	5.85	2.07	5.00	6.00
28	坚硬	hardy	5.24	1.59	4.38	2.03	4.98	1.90	5.66	1.94	7.00	12.00
29	考究	well-dressed	5.38	1.65	4.34	2.00	5.28	1.83	4.68	2.07	6.00	7.00
30	厉害	badly	4.90	1.78	5.54	1.89	5.08	1.89	5.53	1.93	5.00	10.00

World List 5

No.	Chinese Version	English Transation	Affect		Excitement		Dominance		Familarity		strokes in the first character	strokes in the last character
			M	*SD*	*M*	*SD*	*M*	*SD*	*M*	*SD*		
1	辩护	defend	5.20	1.32	5.23	1.68	5.42	1.64	4.60	2.07	16.00	7.00
2	督促	urger	4.76	1.61	4.47	1.74	5.33	1.80	5.10	1.86	13.00	9.00
3	观望	hesitate	4.91	1.62	4.08	1.57	5.94	1.53	4.88	1.85	6.00	11.00
4	减肥	reduce-weight	4.99	1.68	4.62	2.21	6.15	1.87	5.79	2.21	11.00	8.00
5	评估	assess	4.99	1.41	3.89	1.55	5.03	1.76	5.00	2.01	7.00	7.00
6	帮主	chief	5.09	2.12	4.73	2.06	3.79	1.74	3.45	2.16	9.00	5.00
7	法制	legality	5.00	1.54	4.68	1.77	3.01	1.50	5.31	2.04	8.00	8.00
8	皇帝	emperor	5.17	1.64	5.34	2.20	2.70	1.79	3.81	2.33	9.00	9.00
9	军队	army	4.97	1.67	5.69	1.90	2.92	1.50	4.59	2.16	6.00	4.00
10	脸色	complexion	4.78	1.64	4.42	2.01	6.07	1.65	6.07	1.82	11.00	6.00
11	彻底	thoroughly	5.38	1.51	4.43	1.90	5.01	1.70	5.18	2.03	7.00	8.00
12	纯粹	purely	5.21	1.30	4.05	1.71	5.37	1.73	4.62	1.80	7.00	14.00
13	粗壮	stockily	4.95	1.46	4.52	1.81	5.21	1.71	5.20	1.91	11.00	6.00
14	骄傲	proudly	4.94	1.93	4.86	1.95	6.00	1.55	5.72	1.83	9.00	12.00
15	离奇	quaintly	4.79	1.76	6.05	1.90	4.55	1.81	4.21	1.68	10.00	8.00
16	认错	apology	5.09	1.61	4.78	1.87	5.83	1.94	5.70	1.91	4.00	13.00
17	搜寻	seek	4.94	1.66	5.06	1.90	5.65	1.67	5.28	1.99	12.00	6.00
18	同情	sympathize	5.22	1.66	4.09	1.85	6.25	1.65	5.95	1.93	6.00	11.00
19	挖掘	dig	5.14	1.63	4.78	1.78	5.44	1.64	4.53	1.87	9.00	11.00
20	主宰	decide	4.74	2.02	4.88	1.95	4.51	2.00	4.39	1.87	5.00	10.00
21	农村	village	4.99	1.63	3.89	1.88	5.41	1.97	5.99	2.00	6.00	7.00
22	事物	object	5.17	1.27	3.01	1.71	5.55	1.86	5.63	2.13	8.00	8.00
23	消防	fire-fighting	5.06	1.63	4.59	1.81	4.57	1.85	4.94	1.86	10.00	6.00
24	遗产	legacy	5.26	1.91	5.74	2.18	4.85	2.32	4.34	2.06	12.00	6.00
25	职责	duty	5.27	1.55	4.84	1.92	4.99	1.83	5.35	1.82	11.00	8.00
26	内向	bashfully	4.66	1.44	3.61	1.62	5.40	1.83	6.03	1.67	4.00	6.00
27	谦卑	humbly	4.99	1.84	4.24	1.88	5.40	1.93	5.03	1.89	12.00	8.00
28	深入	deeply	5.44	1.47	4.19	1.72	5.66	1.41	4.89	2.18	11.00	2.00
29	威严	imperially	5.20	1.74	5.17	1.98	3.98	1.91	4.74	1.85	9.00	7.00
30	早熟	prematurely	5.06	1.38	4.81	1.96	5.15	1.74	5.55	1.78	6.00	15.00

* Both wordlist 4 and wordlist 5 were 30-word lists (10 verbs, 10 nouns, and 10 adjectives) that were identical in regard to terms relating to affect ($M4 = 5.01$, $SD4 = 0.18$; $M5 = 5.05$, $SD5 = 0.19$; $t(58) = -0.74$, $p > 0.05$), excitement ($M4 = 4.67$, $SD4 = 0.65$; $M5 = 4.62$, $SD5 = 0.65$; $t(58) = 0.29$, $p > 0.05$), dominance ($M4 = 4.98$, $SD4 = 1.02$; $M5 = 5.04$, $SD5 = 0.94$; $t(58) = -0.25$, $p > 0.05$), familiarity ($M4 = 4.97$, $SD4 = 0.70$; $M5 = 5.06$, $SD5 = 0.66$; $t(58) = -0.51$, $p > 0.05$), strokes of the first character ($M4 = 8.47$, $SD4 = 2.97$; $M5 = 8.83$, $SD5 = 2.87$; $t(58) = -0.49$, $p > 0.05$), and strokes of the last character ($M4 = 8.23$, $SD4 = 3.17$; $M5 = 8.20$, $SD5 = 2.92$; $t(58) = 0.04$, $p > 0.05$).
